# ACL reconstruction for all is not cost-effective after acute ACL rupture

**DOI:** 10.1136/bjsports-2020-102564

**Published:** 2021-03-18

**Authors:** Vincent Eggerding, Max Reijman, Duncan Edward Meuffels, Eline van Es, Ewoud van Arkel, Igor van den Brand, Joost van Linge, Jacco Zijl, Sita MA Bierma-Zeinstra, Marc Koopmanschap

**Affiliations:** 1 Orthopedics, Erasmus Medical Center, Rotterdam, Zuid-Holland, The Netherlands; 2 Orthopedics, Medisch Centrum Haaglanden, Den Haag, Zuid-Holland, The Netherlands; 3 Orthopedics, Elisabeth-TweeSteden Ziekenhuis, Tilburg, Noord-Brabant, The Netherlands; 4 Orthopedics, Reinier de Graaf Gasthuis, Delft, Zuid-Holland, The Netherlands; 5 Department of Orthopaedic Surgery, Sint Antonius Hospital, Nieuwegein, The Netherlands; 6 Department of General Practice and Orthopedics, Erasmus Medical Center, Rotterdam, Zuid-Holland, The Netherlands; 7 Institute for Medical Technology Assessment (iMTA), Erasmus University Rotterdam, Rotterdam, Zuid-Holland, The Netherlands

**Keywords:** anterior cruciate ligament, knee surgery, knee injuries, exercise rehabilitation, sports rehabilitation programs

## Abstract

**Objectives:**

To conduct a cost-utility analysis for two commonly used treatment strategies for patients after ACL rupture; early ACL reconstruction (index) versus rehabilitation plus an optional reconstruction in case of persistent instability (comparator).

**Methods:**

Patients aged between 18 and 65 years of age with a recent ACL rupture (<2 months) were randomised between either an early ACL reconstruction (index) or a rehabilitation plus an optional reconstruction in case of persistent instability (comparator) after 3 months of rehabilitation. A cost-utility analysis was performed to compare both treatments over a 2-year follow-up. Cost-effectiveness was calculated as incremental costs per quality-adjusted life year (QALY) gained, using two perspectives: the healthcare system perspective and societal perspective. The uncertainty for costs and health effects was assessed by means of non-parametric bootstrapping.

**Results:**

A total of 167 patients were included in the study, of which 85 were randomised to the early ACL reconstruction (index) group and 82 to the rehabilitation and optional reconstruction group (comparator). From the healthcare perspective it takes 48 460 € and from a societal perspective 78 179 €, to gain a QALY when performing early surgery compared with rehabilitation plus an optional reconstruction. This is unlikely to be cost-effective.

**Conclusion:**

Routine early ACL reconstruction (index) is not considered cost-effective as compared with rehabilitation plus optional reconstruction for a standard ACL population (comparator) given the maximum willingness to pay of 20 000 €/QALY. Early recognition of the patients that have better outcome of early ACL reconstruction might make rehabilitation and optional reconstruction even more cost-effective.

## Introduction

ACL rupture is one of the most common injuries in the young athlete. For patients after ACL rupture, knee-related quality of life (QoL) is impaired for more than 20 years compared with population norms, and even more when compared with peers.^1 2^ Not only for the individual, but also from a societal perspective ACL rupture has a huge impact. The number of ACL ruptures and reconstructions are increasing. In the past 15 years, the number of ACL reconstructions in the Netherlands increased with over 130% from around 3600 reconstructions in 2003 to over 8400 in 2018.^3 4^ This increase in number of reconstructions leads to an increased socioeconomic burden.

Treatment options after ACL rupture are an early ACL reconstruction, or a rehabilitation and optional reconstruction in case of persistent instability. Both treatments can lead to comparable clinical results and do not show a difference in the occurrence of post-traumatic osteoarthritis.^5–7^ ACL surgery does restore objective physical stability and might in that way prevent secondary injuries. On one hand, rehabilitation and optional reconstruction after this rehabilitation period is more uncertain for patient and surgeon with the risk of recurrent instability and delayed reconstruction versus an early ACL reconstruction. On the other hand in case of early ACL reconstruction, such surgery has the risk of complications as among others stiffness (1%–4%), septic arthritis (0.1%–1.7%), deep venous thrombosis (0.53%–14.9%) and re-rupture of the graft (3.2%–11.1%),^8 9^ while a part of the patients would not have needed this surgery when they had tried rehabilitation first.

With the increasing healthcare costs and comparable clinical outcome of different medical treatments, value calculations are becoming increasingly important.^10^ They provide essential information for patients, physicians and policy makers in healthcare to support their decisions. Cost-utility analysis might help clinicians in deciding on what treatment options produce most health, given the necessary costs. This cost-utility study shows what happens with QoL and costs if care as usual: rehabilitation plus optional reconstruction would be replaced by early reconstruction for this specific patient group. In other terms, we will study the gains/losses in health-related QoL and changes in costs when switching from care as usual to early reconstruction. If a QoL gain is observed, the analysis shows how much we have to pay for each additional quality-adjusted life year (QALY).

Several studies have been published on the costs of ACL reconstruction with different grafts and with a decision-tree analysis for competitive athletes, but so far not with the use of real patient-data of a randomised controlled trial.^11–13^

The aim of this study was to evaluate the cost-utility of early ACL reconstruction (index) versus rehabilitation plus an optional reconstruction after acute ACL rupture (comparator) with the use of data of a randomised controlled trial.

## Methods

This cost-utility study was performed with the data of the Conservative vs Operative Methods for Patients with ACL Rupture Evaluation (COMPARE) study, an open-label randomised controlled trial for patients after an acute ACL rupture. Patients were randomised to an early ACL reconstruction (index), or rehabilitation plus an optional reconstruction in case of recurrent instability after a rehabilitation period of 3 months (comparator). For a full description of the study and results, we refer to the clinical outcome study.^14^

Briefly, data on QoL, medical costs and productivity costs were collected through patient questionnaires performed at baseline, and 3, 6, 9, 12 and 24 months follow-up.^15–17^

Cost-utility was calculated as incremental costs per QALY gained of early ACL reconstruction (index) compared with rehabilitation plus optional reconstruction (comparator), using two perspectives: the healthcare system perspective and the societal perspective.^18^

We used the 3-Level EuroQol Questionnaire (EQ-5D-3L) to assess QoL.^19^ The EQ-5D-3L covers five dimensions: mobility, self-care, usual activities, pain/discomfort and anxiety/depression. Each dimension has three levels: no problems, some problems and extreme problems. The outcome score is between 1 (best QoL) and 0 (very poor comparable to death), and a normative value for persons aged 30–39 is 0.901. Some health states can be considered as even worse than death and therefore even negative values are possible.^20^

Over the period of 2 years, the difference in area under the curve of the QoL between-groups was calculated to determine the QoL gain per year in QALY. QoL was measured on the following moments after randomisation: 0, 3, 6, 9, 12 and 24 months. QALYs were calculated as follows as area under the curve: QALYs year 1=[(q0+q3)/2+(q3+q6)/2+(q6+q9)/2+(q9+q12)/2]/4

QALYs year 2=(q12+q24)/2

Total QALYS over 2 years=QALYs year 1+QALYs year 2

From the healthcare system perspective, medical costs related to knee problems were included: cost of hospital care (including incremental imaging, surgery and outpatient clinics visits), non-hospital care (such as physical therapy, general practitioners care) and medication use with the use of the Institute for Medical Technology Assessment(iMTA) Medical Consumption Questionnaire.^15^

From the societal perspective, both medical and non-medical costs related to knee problems were included. Non-medical costs refer to productivity costs related to paid work (due to absence from work because of knee-related problems, using the friction cost method and/or reduced productivity at work) and costs related to a lower ability to perform unpaid activities because of knee-related problems (such as household tasks) with the use of the iMTA Productivity Cost Questionnaire.^15^ Other non-medical costs refer to travel costs to and from hospitals and suppliers of community care. Non-medical costs were calculated using the most recent Dutch guidelines for economic evaluation studies in healthcare.^17^ Costs were valued in euros for the year 2018. For the second year, costs and health effects were discounted: costs by 4% and QALYs by 1.5% conform the Dutch guidelines for economic evaluation in healthcare.^21^

### Analysis

Patients were analysed according the intention-to-treat principle. Missing values for costs and/or QoL were imputed, based on linear interpolation in case the amount of missing values was less than 20%. Adjustments for baseline values would have used if there were relevant differences in baseline characteristics among the study groups. Costs and QALYs were summed over the 24 months study period using the information of all follow-up moments.

The uncertainty for costs and health effects was assessed by means of non-parametric bootstrapping, in which 5000 observations were randomly drawn from the available study.^22^ The incremental costs and health effects for each bootstrap sample were displayed on a cost-effectiveness plane. An acceptability curve was drawn to indicate the probability that the cost-effectiveness ratio is acceptable, given various thresholds for the maximum willingness to pay for one QALY gained.

## Results

### Patients

Baseline characteristics are presented in table 1, and did not differ among the study groups. In the randomised controlled trial, 167 patients were included, of which 85 were randomised to the early ACL reconstruction (index) and 82 to the rehabilitation plus optional reconstruction. Of the 85 patients randomised to early ACL reconstruction (index), 3 patients were not reconstructed; one because of tomophobia and two because of negative instability testing under anaesthesia. Of the 82 patients treated with rehabilitation and optional reconstruction, 41 patients (50%) eventually received reconstruction surgery during 2-year follow-up. Follow-up rates were considered high with 98% among the different groups. The amount of missing values among the cost and QoL data during follow-up was only 6%.

**Table 1:** Baseline characteristics

	Early reconstruction (n=85)	Rehabilitation plus an optional reconstruction (n=82)
Age, years	31.2 (±10.3)	31.4 (±10.7)
Female, no. (%)	36 (42.4)	31 (37.8)
Body mass index, kg/m^2^	24.3 (±3.7)	25.0 (±4.1)
Tegner score (0–10)	7.0 (±2.3)	7.1 (±2.0)
College education, no. (%)	30 (35.3)	36 (43.9)
Paid work, no. (%)	71 (83.5)	64 (78.0)
EQ-5D-3L	0.74 (±0.20)	0.75 (±0.21)

Mean and SD within parentheses or reported otherwise.

EQ-5D-3L, 3-Level EuroQol Questionnaire.

### Quality of life

**Figure 1:**
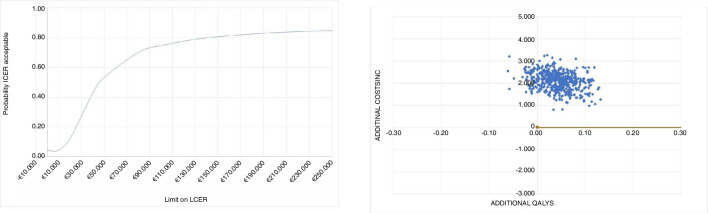
Quality of life (EQ-5D) on the different time points (months).

For the period of 24 months, patients in both treatment arms experience a QoL between 0.72 and 0.84. Patients treated with an early reconstruction (n=85) have a total of 1.73 (SD 0.20) QALY and patients treated with rehabilitation plus an optional reconstruction (n=82) have a total of 1.69 (SD 0.21) QALY during the study period. On average, patients treated with an early reconstruction have a slightly better QoL, as the difference is about 0.04 QALYs over the course of 2 years, see figure 1 (p value=0.18).

### Costs

Healthcare system costs were 6368 € (SD 1630 €) in the early reconstruction group and 4267 € (SD 3011 €) in the rehabilitation plus optional reconstruction group. Productivity costs were 8489 € (SD 9659 €) in the early reconstruction group and 7214 € (SD 9137 €) in the rehabilitation plus optional reconstruction group, see table 2. Productivity costs due to paid work vary substantially across patients in both arms (see large SDs in table 2).

**Table 2:** Average costs per patient per treatment arm in euros

	Early reconstruction (n=85)	Rehabilitation plus an optional reconstruction (n=82)
*Healthcare system*		
Hospital costs (SD)	4348 (1130)	2526 (1947)
Extramural costs		
Sports medicine	23	44
General practitioner	16	18
Occupational medicine	33	19
Physical therapist	1931	1650
Sum extramural	2003 (1166)	1731 (1386)
Medication	16	10
1. Total costs from healthcare system perspective (SD)	**6367 (1630)**	**4267 (3011)**
*Societal*		
Absence paid work (SD)	5636 (7549)	4448 (6987)
Presenteeism paid work (SD)	1480 (2931)	1262 (2624)
Unpaid work (SD)	1373 (2636)	1504 (3045)
2. Productivity costs total (SD)	**8489 (9659)**	**7214 (9137)**
3. Direct non-medical costs (travel costs)	**94**	**79**
Total costs from societal perspective (1+2+3)	**14 951** **(10 004)**	**11 558** **(10 579)**

### Cost-utility

Table 3 shows the results of the cost-utility analysis for both treatment regiments. Applying the healthcare system perspective it takes 48 460 € to gain a QALY when performing early reconstruction instead of rehabilitation plus an optional reconstruction.

**Table 3:** Cost-utility results of early reconstruction versus rehabilitation plus optional reconstruction

	Healthcare system perspective	Societal perspective
Incremental cost (in €)	2101	3393
Incremental QALYs	0.043	0.043
Incremental cost per QALY (ICER in €)	48 460	78 179

ICER, incremental cost-effectiveness ratio of early ACL reconstruction (index) versus rehabilitation plus an optional reconstruction in case of recurrent instability (comparator); QALY, quality-adjusted life year.

With the iMTA disease burden calculator, we estimated patients loss of QALY around 5% compared with healthy peers, which is estimated as a low burden of disease.^23^ Given this low burden of disease patients experience after ACL rupture, the maximum willingness to pay in the Netherlands would be up to 20 000 €/QALY according to the Dutch Healthcare Institute.^10^ The uncertainty analysis (bootstrapping) indicates that the probability that the cost-utility meets this standard is 12%. In case of a threshold of 50 000 € per QALY gained, this probability is 54%.

**Figure 2:**
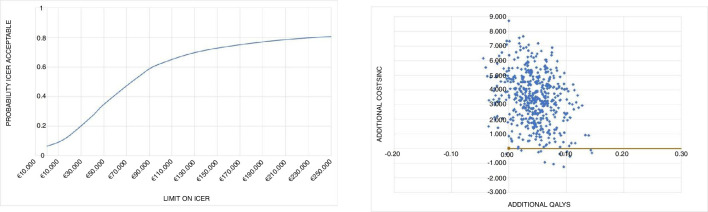
Cost-effectiveness plane and acceptability curve from the healthcare system perspective. ICER, incremental cost-effectiveness ratio. Costs in euros and valued for 2018.

**Figure 3:**
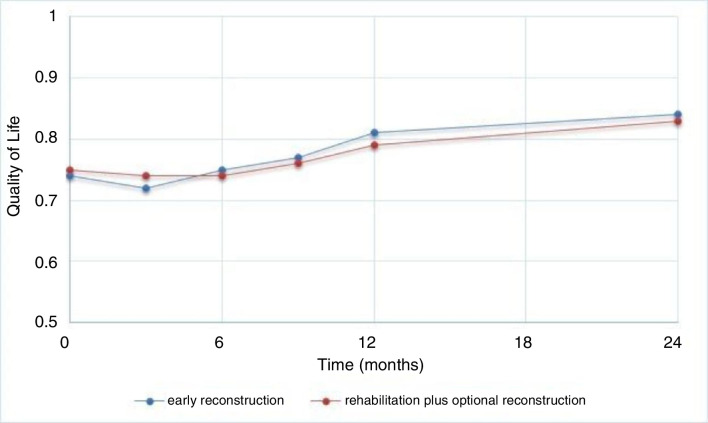
Cost-effectiveness plane and acceptability curve from the healthcare system perspective. ICER, incremental cost-effectiveness ratio. Costs in euros and valued for 2018.

Using the societal perspective it takes 78 179 € to gain a QALY when performing early reconstruction compared with rehabilitation plus an optional reconstruction. In figure 2, the results of the cost-utility are depicted. Early reconstruction led to better QoL in 90% of bootstrap replications (right side of the diagram); in 92% of the replications early reconstruction led to a more expensive treatment (upper right quadrant). Almost all of the 10% patients with a worse QoL were more costly (upper left quadrant). The uncertainty analysis gives a 14% probability that incremental costs are lower than 20 000 € per QALY. In case of a threshold of 50 000 € per QALY gained, this probability is 35%. This is illustrated in the acceptability curve from the societal perspective (figure 3).

## Discussion

We are the first to analyse the cost-utility of two commonly used treatments for patients after ACL rupture with the use of actual patient data from a randomised controlled trial. Both treatments resulted in a relatively good QoL.^1^ Patients treated with an early ACL reconstruction (index) experienced a slightly higher QoL over the observed 24-month period. On the other hand, early ACL reconstruction (index) leads to higher costs (both medical and non-medical). This resulted in a cost-utility ratio of 48 460 €/QALY from the healthcare system perspective and 78 179 €/QALY from the societal perspective.

As the QoL of these patients is relatively good, the burden of disease is limited.^1^ Health-related QoL after ACL rupture is even better compared with a general population, probably due to the fact that ACL rupture is more common in healthy and active individuals.^1^ Given the low burden of disease patients experience after ACL rupture, the maximum willingness to pay would be up to 20 000 €/QALY in the Netherlands, according to the advice of the Dutch Healthcare Institute.^24^ Uncertainty analysis gives a 12% probability for the healthcare system perspective and 14% for the societal perspective to meet this criterion, which is considered low. Therefore it is unlikely for early ACL reconstruction (index) to be cost-effective, compared with rehabilitation plus optional reconstruction, according to Dutch policy standards.

The early ACL reconstruction not being cost-effective is mostly caused by the low difference in QALY of 0.04 between both groups. This low difference in QoL is in line with other clinical outcome measures used in the clinical study (among others the International Knee Documentation Committee Score, Knee Injury and Osteoarthritis Outcome Score and the Lysholm), and therefore we are confident that the difference between groups is not underestimated.^14^ Also, QALYs are population dependent and in a different population the difference between both treatments might differ.

In the rehabilitation plus an optional reconstruction in case of recurrent instability (comparator) group there are two distinct groups; patients who perform well with rehabilitation alone and patients with a delayed reconstruction. Patients who succeed with rehabilitation alone have the highest mean QALY (of 1.74 over 2 years) and lowest mean healthcare system costs of 1988 € and mean societal costs per patient of 7223 €. Patients treated with a delayed ACL reconstruction have the lowest mean QALY (of 1.64 over 2 years) and highest mean healthcare system costs of 6656 and mean societal costs of 16 111 € per patient.

If we are able to discriminate patients at an early stage that perform well with rehabilitation from those who do not, it is likely we decrease costs even more by reducing the number of patients who have two rehabilitation programmes; one before the reconstruction and one after.

To estimate the willingness to pay for a specific condition is an ethical and political issue. We used the recommended method for calculating the burden of disease (proportional shortfall). A limitation of this method is that it only partially takes into account the patients’ age. One could argue that a younger patient has more value on an ACL reconstruction, because he has more active life years to go.^25^ But, given the relatively good QoL these patients still have, it is still unlikely that direct reconstruction would then be considered cost-effective. Also we might be more reserved with an early ACL reconstruction in younger patients, because young and active patients have the highest risk of a new knee injury possibly and the young might be helped more in the long run with adjustment of their activity level.^8^

Von Essen *et al* 13 found that acute reconstruction resulted in less sick-leave days and fewer indirect costs to the individual and society and was cost-effective. But they did not take into account that 50% of patients treated with a rehabilitation programme are doing well with this treatment and were not reconstructed after all.

Strengths of our study are the use of the largest multicenter randomised controlled trial for patients after ACL rupture, a high 2-year follow-up rate of 98% and clear analysis of cost-utility by the latest standards.

Possible limitations of our study include the broad inclusion criteria: we used broad inclusion criteria (eg, 18–65 years of age and patients with all activity levels) and analysed the groups as a whole. This leads to high level of generalizability, but might take away differences for certain subgroups. It is likely that in selected patient groups the procedure will be more cost-effective as, for example, in the study of Stewart *et al* found a cost-utility ratio of $22 702 per QALY gained in competitive athletes with the use of a decision-tree analysis.^12^

Another limitation is the variability in costs as seen in the high SD especially in the societal costs and the relatively small patient numbers to perform the cost-utility analysis. Furthermore, for the calculation of QoL we used the EQ-5D-3L questionnaire, which was the standard questionnaire at the start of the study. Nowadays the EQ-5D-5L is the standard and this questionnaire is higher in responsiveness in patients with high QoL.^26^ Nevertheless, the EQ-5D-3L we used has shown in general to be capable to detect meaningful differences in QoL.^26^

In conclusion, an early ACL reconstruction (index) leads to a 0.04 increase in QALY over a period of 2-year compared with rehabilitation plus an optional reconstruction, but is with a cost of 48 460 €/QALY (healthcare system perspective) to 78 179 €/QALY (societal perspective) not considered cost-effective given the maximum willingness to pay of 20 000 €/QALY for routine practice.

**What are the findings?**

- Patients after ACL rupture treated with an early ACL reconstruction experienced a slightly higher quality of life over the observed 24-month period compared with patients treated with rehabilitation and optional reconstruction.
- The small difference in quality of life and substantial higher costs are unlikely to make early ACL reconstruction for all cost-effective.
- Patients who fail non-operative treatment and undergo consequential ACL reconstruction have the lowest quality of life and highest costs.

**How might it impact on clinical practice in the future?**

- Our study shows the importance to know the most optimal treatment for the individual patient as soon as the diagnosis has been made. Future research should focus on early identification of the what the most optimal treatment will be for the individual patient.

## Data Availability

Data are available upon reasonable request. Data may be obtained from a third party and are not publicly available. We agree on data sharing to British Journal of Sports Medicine when required according to the World Health Organization and Nordic Trial Alliance declaration about clinical trial transparency.
